# Calcium Medronate-Based Metal–Organic Frameworks as Multifunctional Biomaterials

**DOI:** 10.1021/acs.cgd.4c01478

**Published:** 2025-02-20

**Authors:** Pablo Salcedo-Abraira, María Fernández-Grajera, Francisco A. Guerrero-Román, Antonio Rodríguez-Diéguez, Veronica Luque-Agudo, María Luisa González-Martín, Amparo M. Gallardo-Moreno, Sara Rojas

**Affiliations:** † Department of Inorganic Chemistry, 16741 University of Granada, Avda. Fuente nueva s/n, 18071 Granada, Spain; ‡ Center for Networked Biomedical Research on Bioengineering, Biomaterials and Nanomedicine (CIBER-BBN), Badajoz 06006, Spain; § Department of Natural Systems and Resources, School of Forest Engineering and Natural Resources, 16771 Polytechnic University of Madrid, C/José Antonio Novais, 10, 28040 Madrid, Spain; ∥ Department of Applied Physics, Faculty of Sciences, University of Extremadura, Badajoz 06006, Spain; ⊥ University Institute of Biomedical Research (INUBE), Badajoz 06006, Spain

## Abstract

Metal–organic frameworks (MOFs) can be prepared from bioactive molecules, which are released during the degradation of the material in the body. Particularly, MOFs have recently emerged as bisphosphonate (BP) drug delivery systems. In this work, two novel MOFs based on the smallest bisphosphonate medronic acid (MA) and calcium with formulas [Ca­(CH_4_O_6_P_2_)·H_2_O] (GR-MOF-23) and [Ca­(CH_4_O_6_P_2_)·CH_3_OH] (GR-MOF-24) in aqueous and/or methanolic solutions at room temperature were synthesized and fully characterized. The stability test performed in simulated physiological conditions (a phosphate buffer saline (PBS, pH = 7.4, 10 mM) solution at 37 °C) showed a progressive Ca^2+^ leaching from both GR-MOF-23 and GR-MOF-24, achieving 38.0 ± 2.8 and 35.8 ± 3.9% release of calcium after 1 week of suspension. Interestingly, the recovered solid residues from the stability tests were identified as apatite and calcium phosphate phases, which might facilitate the formation of bone apatite and collagen. The antibacterial activity of GR-MOF-23 and GR-MOF-24 was investigated against Escherichia coli and Staphylococcus aureus, among the most relevant human pathogens, causing a wide variety of infections in bone fracture in osteoporosis and prosthesis. While both materials exhibited bacteria growth inhibition, GR-MOF-24 also showed a bactericide action, likely due to a more progressive release of Ca^2+^, which is the ion related to the improved stability of the biofilm. These innovative materials present exciting opportunities for developing antibacterial surfaces in prosthetics and the treatment of bone fracture infections.

## Graphical abstract

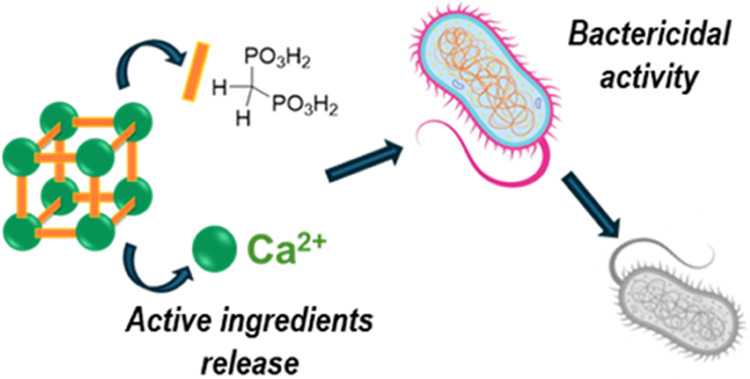

## 1 Introduction

Osteoporosis (meaning “porous bone”) is a disease characterized by low bone mass and microarchitectural deterioration of bone tissue, leading to increased vulnerability of fractures and bone fragility. Fractures are a significant health complication of osteoporosis, contributing to higher mortality rates, disability, loss of independence, and increased healthcare expenses.^1^ Considering the worldwide aging population, the importance of the prevention and management of osteoporotic fragility fractures is increasing with time. It is estimated that more than 200 million people worldwide suffer from osteoporosis.^2^ According to the International Osteoporosis Foundation, 43% of women and 29% of men over the age of 50 years experience an osteoporosis fracture (a broken bone following a minor fall or bump).^3,4^ Aside from pain, fragility fractures are associated with morbidities, need for long-term care, disabilities, and mortality. In this regard, the direct annual cost of treating osteoporotic fractures of people on average is reported to be between US$5000 and 6500 billion in Canada, Europe, and the United States alone, not considering indirect costs such as disability and loss of productivity.^5^

Other complications associated with bone fracture in osteoporosis or even in prostheses must also be considered. In this scenario, the weakened structure of osteoporotic bone facilitates bacterial infections,^6^ especially in those cases where the bone is reconstructed or replaced by an implant. Further, infection of prostheses is one of the problems most feared by orthopedic surgeons. In these cases, bacteria may colonize the tissue and the biomaterial in the fracture, causing osteomyelitis, which complicates healing and requires longer treatment. Therefore, treatments that face osteoporosis but also effectively prevent possible bacterial colonization are very promising.

In all recently published guidelines on the treatment of osteoporosis, calcium supplements or calcium/vitamin D combinations are currently recommended as co-medications with antiresorptive therapy.^7^ Calcium is a major building block of bone (99% of the skeleton) that acts as a reservoir for maintaining calcium levels in blood needed for healthy nerves and muscles. Also, Ca plays an important role in collagen synthesis, enhancing the cross-linking of the collagen molecules when introduced between them.^8^ On the other hand, numerous pharmacological therapies based on bisphosphonates (BPs) have been proposed to reduce the fracture risk in patients with osteoporosis. The two phosphonic acids present in the molecular structure cause BPs to be avidly adsorbed onto the surface of apatite crystals in bone, primarily at sites of active bone remodeling.^9^ Furthermore, different BPs have shown some antibacterial effects.^10,11^ In a way to improve the activity of BPs, they have been incorporated in coatings, enabling the direct delivery of these drugs in a local area, which will precisely enhance osteointegration and bone repair without the systemic side effects.^12^ Porous materials, which have been investigated as drug delivery systems, may provide a more advantageous alternative to achieve controlled and localized delivery. However, the low efficiency achieved in BP loading is a major challenge, as many drugs remain in the solution without entering the mesoporous interior.

Here, a combined formulation with both BPs and calcium is proposed as an innovative approach for providing both active ingredients in a simple formulation. Among all of the combined formulations, metal–organic frameworks (MOFs) have recently emerged as BP drug delivery systems.^13,14^ MOFs represent an interesting family of hybrid materials based on metal ions interconnected through organic polydentate linkers, giving rise to an ordered structure of channels and cavities accessible to guest molecules.^15^ BPs are excellent linkers for the construction of MOF materials, as they possess at least two complex-forming groups that enable their coordination to cations. The release of the active ingredients is then achieved through the degradation of the framework under biological conditions,^16^ being the release affected by the nature of the functional groups of the BPs.^17^ In this sense, medronic acid (MA) is the simplest BP that exists, with no additional functional groups or an alkyl chain. As far as we know, fewer than a dozen studies reported the potential of BPs as linkers in MOFs and their application in osteoporosis. One of the first MOFs based on BPs as linkers is BioMIL-4, a material from the Institut Lavoisier.^18^ This biocompatible MOF based on Ca^2+^ and alendronate was demonstrated to be inert in contact with biological simulated fluid due to its very high stability. Then, Rocha et al. described two MOFs (named CaP1 and CaP2) based on *p*-xylylenebisphosphonate and Ca^2+^.^19^ In particular, CaP1 was not toxic and stimulated bone mineralization when tested in MG63 osteoblast-like cells. Then, Michaelis et al. reported the isostructural Sr^2+^ and heterometallic Sr^2+^/Ca^2+^ containing derivatives of CaP1 as controlled metal delivery systems along with the ability to interact with albumin.^20^ Then, Demadis et al. reported a series of biocompatible six MOFs based on etidronate, alendronate, pamidronate, neridronate and Ca^2+^, and alendronate and neridronate and Mg^2+^.^21^ Recently, the same group successfully synthesized two new materials based on Ca^2+^ and Sr^2+^ and risedronate in a way to improve the solubility of risedronate.^16^ In another work, Almeida Paz et al. described a family of MOFs based on alendronate and Mg^2+^ and mixtures of Mg^2+^ and Ca^2+^, which promote osteoblast metabolic activity.^22^ Thus, this article pioneering reports the synthesis and characterization of the first two novel MOFs (namely, GR-MOF-23 and GR-MOF-24) based on the bisphosphonate MA and Ca^2+^, evaluating some *in vitro* bioactivity (in terms of stability and apatite formation) and antibacterial activity.

## 2 Experimental Section

### 2.1 Materials and Methods

All reagents were purchased from commercial sources and used as received without additional purification. Methylenediphosphonic acid (medronic acid, MA) (98%, Acros Organics), calcium nitrate tetrahydrate (99%, ACS Reagent), calcium carbonate (99%, ACS Reagent), calcium acetate hydrate (94%, Supelco), methanol (99.8%, Labkem), and absolute ethanol (Labkem).

### 2.2 Physicochemical Characterization

Fourier transform infrared (FTIR) spectra were measured in the solid state on Bruker Tensor 27 FTIR in the attenuated total reflectance (ATR) mode in the range of 4000 to 400 cm^–1^, and Opus software was used as the data collection program. Routine powder X-ray diffraction (XRPD) patterns were collected on a Bruker D8 Discover diffractometer equipped with a PILATUS3R 100 K-A detector and using Cu Kα radiation (*λ* = 1.5406 Å). The XRPD patterns were registered with a 2*θ* range from 3 to 50° with a step size of 0.02° and a scan rate of 30 s per step at Centro de Instrumentación Científica of the University of Granada. Thermogravimetric analyses (TGAs) were carried out in a thermogravimetric analyzer mode. TGA/DSC1METTLER-TOLEDO with a general heating profile from 30 to 900 °C with a heating rate of 10 °C·min^–1^ under air using a flux of 100 mL·min^–1^. Elemental analyses (EAs) were carried out on a Thermo Scientific analyzer, Flash 2000. Scanning electron microscopy (SEM) was carried out using a Hitachi S510 microscope at 25 kV coupled with a SE detector of 7 nm at 25 kV. Inductively coupled plasma optical emission spectroscopy (ICP-OES) was done in a PerkinElmer spectrometer Optima 7300DV at Servicios Centrales de Apoyo a la Investigación (SCAI), University of Málaga.

### 2.3 Synthesis of GR-MOF-23

10 mg (0.057 mmol) of MA was dissolved in 1 mL of distilled water. In a separate vial, 13.4 mg (0.057 mmol) of Ca­(NO_3_)_2_·4H_2_O was dissolved in 1 mL of methanol. Then, the ligand solution was mixed with the metallic solution. The final solution was sonicated (3 min) and left under ambient temperature in a closed vial for 48 h. Suitable crystals for single-crystal X-ray diffraction were obtained and filtered off under air. Yield: 37% based on the metal.

### 2.4 Synthesis of GR-MOF-24

10 mg portion (0.057 mmol) of MA was dissolved in 1 mL of methanol. In a separate vial, 9 mg (0.057 mmol) of Ca­(CH_3_COO)_2_·H_2_O was dissolved in 1 mL of methanol. The ligand solution was mixed over the metallic one. The final solution was sonicated (3 min) and left under ambient temperature in a closed vial for 48 h. Suitable crystals for single-crystal X-ray diffraction were obtained and filtered off under air. Yield: 71% based on metal.

### 2.5 Scale-Up Synthesis of GR-MOF-23

The synthesis was scaled up to 10 times. 10 mL of an aqueous solution containing 100 mg (0.57 mmol) of medronic acid was added to 10 mL of a methanolic solution containing 134 mg (0.57 mmol) of Ca­(NO_3_)_2_·4H_2_O. The final solution was sonicated (3 min) and left at ambient temperature in a closed vessel. After 48 h, the obtained white powder was filtered off and washed with water and methanol. Obtained yield: 39% based on metal.

### 2.6 Scale-Up Synthesis of GR-MOF-24

The synthesis was scaled up to 10 times. 5 mL of a methanolic solution containing 100 mg (0.57 mmol) of MA was added to 5 mL of a methanolic solution containing 90 mg (0.57 mmol) of Ca­(CH_3_COO)_2_·H_2_O. The final solution was sonicated (3 min) and left at ambient temperature in a closed vessel. After 48 h, the obtained white powder was filtered off and washed with water and methanol. Obtained yield: 58% based on metal.

### 2.7 Stability Studies

The chemical stability of GR-MOF-23 and GR-MOF-24 in phosphate buffer saline (PBS, pH = 7.4, 10 mM) at 37 °C was evaluated by measuring the release of the constitutive metal (Ca^2+^) by ICP-OES. 20 mg of each material was suspended in 20 mL of PBS under stirring for a week; at different intervals of time (0.25, 0.5, 1, 2, 4, 8 h, 1, 2, 5, and 7 days), the suspension was centrifuged (14,000 rpm, 5 min) and the liquid phase was analyzed by ICP-OES. The structural stability of GR-MOF-23 and GR-MOF-24 of the solid residue was checked by XRPD after being suspended in PBS at 37 °C for 1 week. The stability studies were performed in triplicate (*n* = 3).

### 2.8 Antibacterial Tests

The antibacterial activity was evaluated with the minimum inhibitory concentration (MIC) and minimum bactericidal concentration (MBC) using a microdilution method in 96-well flat-bottomed polystyrene microtiter plates (Greiner bio-one) according to the Clinical and Laboratory Standards Institute guidelines.^23^

The antimicrobial tests were performed with two different bacterial strains, the Gram-positive bacteria, Staphylococcus aureus (ATCC 29213), and the Gram-negative, Escherichia coli (ATCC 25922). From porous beds (Microbank Pro-Lab Diagnostics) at 80 °C, blood agar plates (OXOID Ltd.) were inoculated at 37 °C for 24 h. A bacterial colony was grown in 5 mL of Trypticase Soy Broth (TSB) (OXOID Ltd.) at 37 °C for 9 h. Then, an overnight culture was inoculated with 25 μL of the previous culture. After this time, microorganisms were resuspended in TSB at 82% transmittance at 492 nm using a horizontal light spectrophotometer (Helios epsilon model, Thermo Spectronic, Thermo Fisher Scientific Inc.), and the resulting suspension was diluted 1/100 to obtain a concentration of 10^6^ CFU/mL.

The two novel compounds were diluted in distilled water, obtaining an initial concentration of 33 and 36 mg·mL^–1^ for GR-MOF-23 and GR-MOF-24, respectively. From these stock solutions, 18 concentrations were analyzed starting in the first well from 16.5 mg·mL^–1^ (GR-MOF-23) or 18 mg·mL^–1^ (GR-MOF-24), respectively, and down to 2 mg·mL^–1^.

In each well of the microtiter plate, 100 μL of the prepared bacterial suspension and 100 μL of the materials’ suspensions were mixed, obtaining a final volume of 200 μL. Additionally, positive controls (no compounds added) and negative controls (medium without bacteria) were included. These microtitration plates were incubated for 24 h at 37 °C, with slight agitation. After this time, MIC was determined both visually and with a microplate spectrophotometer reader (ELx800; Bio-Tek Instruments, Inc.). According to standards, the MIC is the first lowest concentration where the bacterial growth is down to 0.09 absorbance.^23^

MBC was also determined by analyzing the bacterial growth on agar plates after inoculating with 20 μL of each of the bacterial suspensions contained in the wells. These agar plates, made from trypticase soy agar (TSA), were incubated for 24 h at 37 °C; after this time, the MBC concentration was determined at the concentration where the bacterial growth was reduced to 99%.

## 3 Results and Discussion

Two novel MOFs, denoted as GR-MOF-23 and GR-MOF-24, were synthesized from calcium and medronic acid (MA) using a simple room-temperature procedure. In brief, GR-MOF-23 and GR-MOF-24 were obtained from a mixture of a methanolic solution of the corresponding calcium salt and a methanolic or aqueous solution of MA at room temperature, reaching yields of 39 and 58%, respectively. Both compounds, with formula [Ca­(CH_4_O_6_P_2_)·H_2_O] (GR-MOF-23, *M*_W_ = 232.08 g·mol^–1^) and [Ca­(CH_4_O_6_P_2_)·CH_3_OH] (GR-MOF-24, MW = 246.11 g·mol^–1^), were prepared in high purity as large single crystals (*ca*. 10 and 1 μm, respectively; Figure 1), suitable for their resolution by single-crystal X-ray diffraction (SCXRD, see Supporting Information Table S1). The crystal structure of GR-MOF-23 comprises two calcium cations with markedly different coordination environments (Figure 2A–C) and is linked by dianionic medronate molecules. Ca1 has a distorted octahedral coordination environment with two axially coordinated water molecules and four monodentated phosphonate groups coordinated in the equatorial plane, with the Ca–O bond distances ranging from 2.326(3) to 2.403(3) Å. Ca2­(II) has a CaO_8_ coordination environment and forms a dimer with a symmetrically equivalent Ca2 atom. The dimeric Ca unit is bridged by two dianionic medronate ligands with Ca···Ca distances of 3.7486(1) Å, where the phosphonate groups have an asymmetric chelating and monodentate coordination, with Ca–O distances ranging from 2.347(3) to 2.748(3) Å depending of the coordination mode. The dimeric calcium units are further connected to other pairs of Ca2 and Ca1 by other two dianionic MA ligands, forming a three-dimensional (3D) structure. O–H···O hydrogen bonds were formed between the oxygens of the phosphonate groups and the coordinated water and the protonated phosphonate groups. When GR-MOF-23 crystals were filtered and left to dry, a novel crystal structure (named GR-MOF-23-dried) was formed. In GR-MOF-23-dried, distorted octahedral Ca­(II) cations are coordinated to six monoanionic monodentated phosphonate groups from different medronate ligands, forming a 3D structure with Ca–O distances ranging from 2.2608(2) to 2.4557(1) Å depending on the phosphonate oxygen atoms (Figure 2D,E). Each monoanionic phosphonate is coordinated to three different Ca­(II) cations, forming a dense 3D framework. The monoanionic phosphonate groups form O–H···O hydrogen bonds with water and the protonated phosphonate groups. Finally, the GR-MOF-24 crystal structure is formed by Ca­(II) cations with the CaO_7_ coordination sphere linked to two monodentated phosphonate groups, two asymmetric chelating phosphonate groups, and a methanol molecule, with Ca–O distances ranging from 2.392(3) to 2.571(3) Å depending on the coordination mode of the phosphonate oxygen atoms (Figure 2F,G). The medronate ligand’s phosphonate group charge is markedly different, having one neutral and one fully deprotonated phosphonate, which overall results in a dianionic medronate. In general, the structure is formed by one-dimensional (1D) chains of calcium atoms linked by dianionic phosphonate groups, which are further expanded into a two-dimensional (2D) framework by the medronate ligands. The 2D layers are packed, forming O–H···O hydrogen bonds with the protonated phosphonate groups and methanol molecules of adjacent coordination polymers.

**Figure 1:**
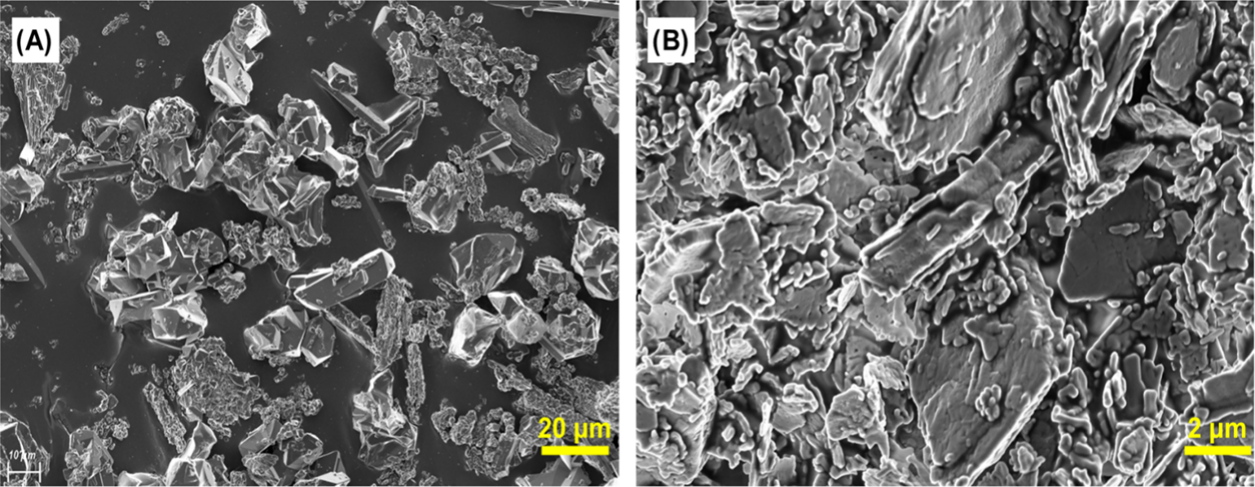
SEM images of (A) GR-MOF-23 and (B) GR-MOF-24.

**Figure 2:**
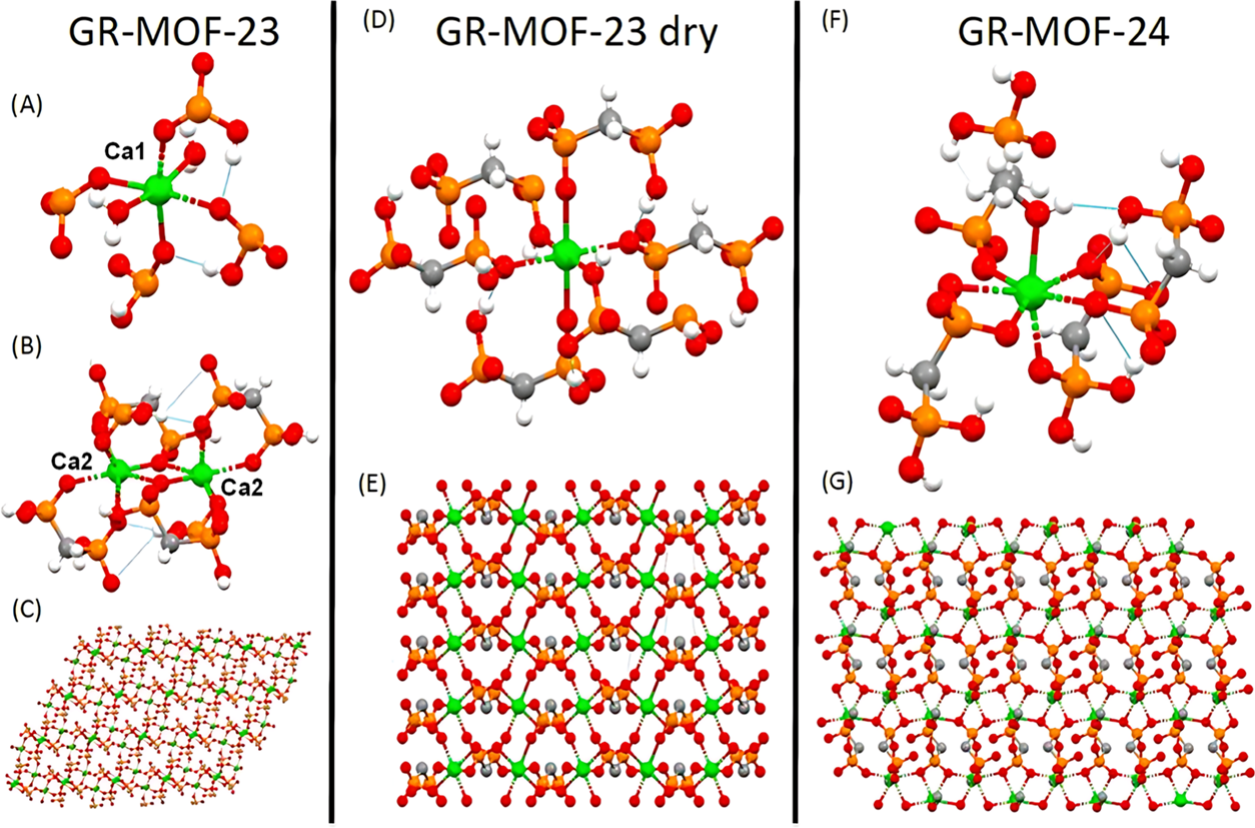
Crystal structure of (A–C) GR-MOF-23; (D, E) GR-MOF-23 dry; and (F, G) GR-MOF-24. Color code: calcium atoms in green, phosphorus in orange, oxygens in red, carbons in gray, and hydrogens in white. Hydrogen bonds are shown as blue lines.

### 3.1 Physicochemical Characterization

It should be noted that the successful scale-up of GR-MOF-23 and GR-MOF-24 from 2 to 20 and 10 mL produces 91.5 and 109.8 mg in a single reaction, respectively. The characteristic crystalline phases of GR-MOF-23 and GR-MOF-24 were identified in the scaled-up bulk by comparing both the location and intensity of the main Bragg reflections with those of the crystalline structure revolved by SCXRD (Figure S1). FTIR spectra show an important shift of the phosphate bands (νP=O, νP–O)^24^ from 1150 and 1204 cm^–1^ in MA, to 1197 and 1177 cm^–1^ for GR-MOF-23, and to 1167 and 1149 cm^–1^ for GR-MOF-24 (Figure S2), confirming the coordination of the phosphate group to the metal centers. Also, for GR-MOF-24, the characteristics of νC–H aliphatic and νO–H of coordinated methanol molecule can be observed at 3250 and 3560 cm^–1^, respectively. Further, thermal stability was evaluated by TGA (Figure S3), where a very small initial loss of weight (2 and 6% for GR-MOF-23 and GR-MOF-24, respectively) from room temperature to 250 °C was observed. These losses were attributed to the departure of some superficial water molecules on GR-MOF-23 and the partial departure of the coordinated methanol molecules on GR-MOF-24. Finally, both materials started to decompose at around 400 °C. Note here that due to the incomplete combustion of the solids and the formation of different calcium phosphates, the corroboration of the molecular formula of the compounds was not possible.

### 3.2 Stability Studies

Considering the importance of calcium in the prevention and treatment of osteoporosis or other bone-related illnesses,^25^ the chemical and structural stability of GR-MOF-23 and GR-MOF-24 in phosphate buffer saline at 37 °C (PBS, 10 mM) was studied. Considering the Pourbaix diagram of Ca at this pH (7.4) and the phosphate concentration (10 mM), the potentially released calcium will be found in the solution as Ca^2+^,^26^ the chemical stability of the prepared compounds was investigated by ICP-OES (Figure 3A). Both materials showed an initial burst release in the first 4 h (29.9 ± 2.3 and 19.9 ± 0.9% for GR-MOF-23 and GR-MOF-24, respectively), followed by a more progressive release and reaching a plateau after 48 h (with 39 ± 3 and 34 ± 2% of total calcium release for GR-MOF-23 and GR-MOF-24, respectively). After one week, these releases were maintained with 38 ± 3 (6.55 mg·g^–1^) and 36 ± 4% (5.83 mg·g^–1^) of released calcium for GR-MOF-23 and GR-MOF-24, respectively. On the other hand, the XRPD patterns showed a total change in the crystalline structures after 1 week in PBS, obtaining similar diagrams for both materials (Figure 4). The nature of the solid residues was identified through Profex software^27^ using the COD database, identifying a mixture of, among others, apatite and calcium phosphate phases (Figure S4). The formation of an apatite residue, together with the release of the active ingredients, might facilitate the formation of bone apatite and collagen, favoring the growth of osteoprogenitor cells on the implant material due to the osteoinductive character of the synthetic apatite.^28^

**Figure 3:**
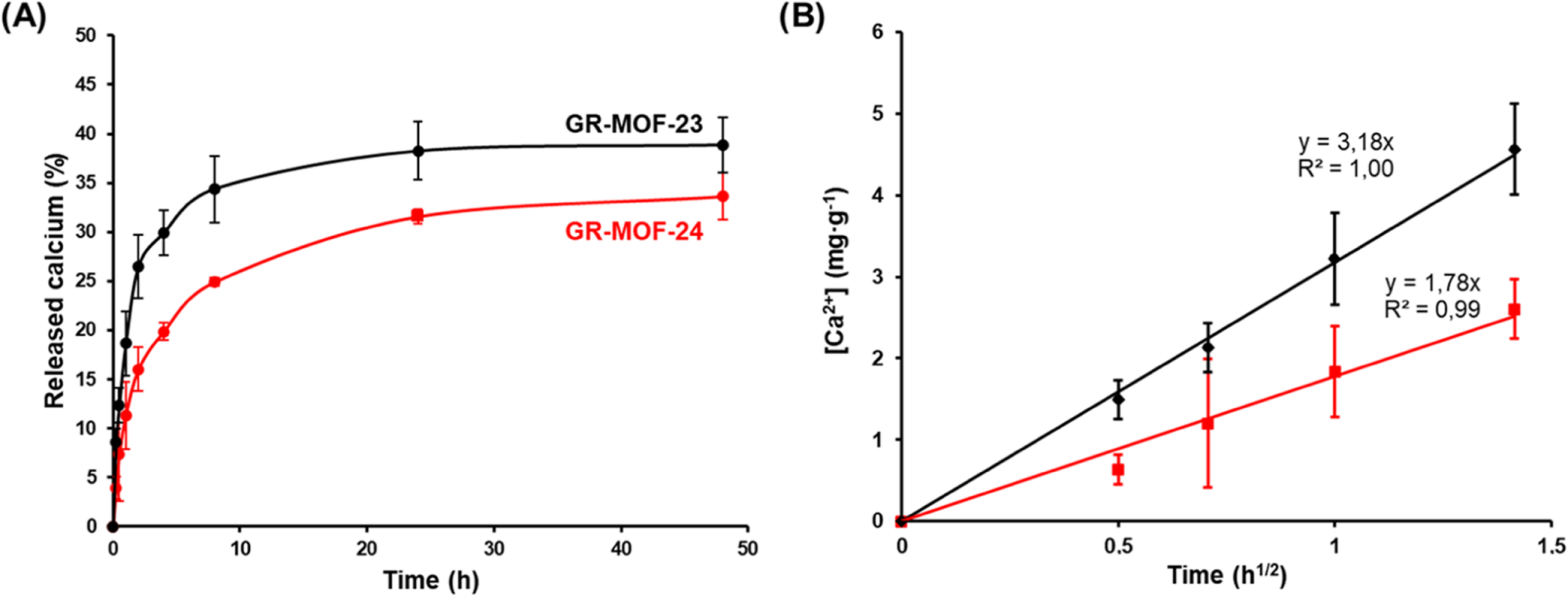
(A) Ca^2+^ leaching over time and (B) fitting to the Higuchi model.

**Figure 4:**
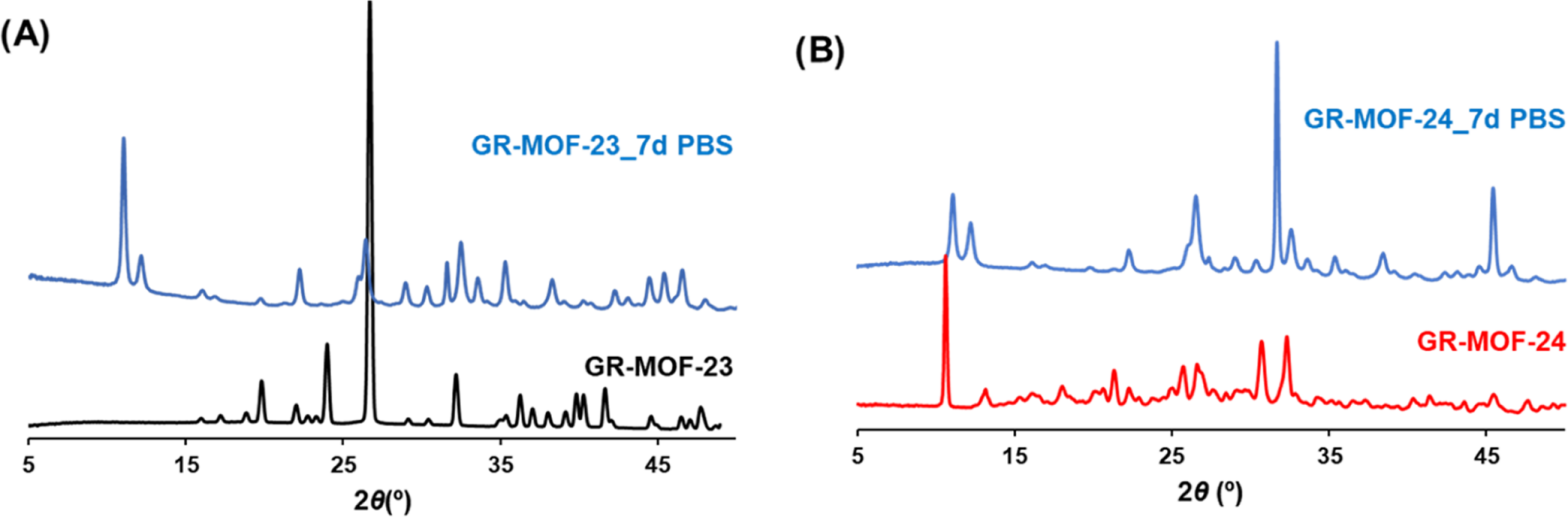
XRPD patterns of (A) GR-MOF-23 and (B) GR-MOF-24 suspended in PBS at 37 °C for 1 week.

In an attempt to compare and understand the involved mechanisms in the Ca^2+^ burst release process, the first two hours of the leaching were fitted to a mathematical model. In particular, the release of Ca^2+^ from both MOFs was fitted to the Higuchi model, which defines the short-time behavior of the release of a dispersed active ingredient from a homogeneous matrix,^29^ and has been used to describe the diffusion of drugs from MOFs.^30^ Despite the differences from the Higuchi’s model and the degradation of a material, and considering that the external diffusion process around the MOF particles is minimized by continuous stirring during the delivery study, the Ca^2+^ release process could be described by the following equation:

$$\left[Ca^{2+}\right]=K\cdot t^{1/2}$$

where [Ca^2+^] corresponds to the concentration of release metal (mg·g^–1^), *t* is the time (h), and *K* is the kinetic constant (g·mg^–1^·h^–1/2^). The Ca^2+^ release from both materials can be empirically adjusted with *R* ^2^ > 0.99 (Figure 3B). The initial delivery rate can be easily compared by estimating the constant diffusion coefficients (3.18 and 1.78 g·mg^–1^·h^–1/2^ for GR-MOF-23 and GR-MOF-24, respectively), observing that GR-MOF-23 is 1.8-fold faster than GR-MOF-24.

### 3.3 Antibacterial Tests

First, the inhibitory character of the materials was evaluated (Table S2). The analysis showed that the MIC values depend more on the composition of the compounds than on the strain used. In this sense, the MIC value of GR-MOF-23 was 16.5 mg·mL^–1^ for both S. aureus and E. coli strains. For GR-MOF-24, these values decreased, with the growth inhibition of 7 mg·mL^–1^ when tested against S. aureus and 7.5 mg·mL^–1^ in the case of E. coli. Interestingly, when these results were compared with the MBC values, it was observed that GR-MOF-24 presented a bacterial effect against S. aureus at 16 mg·mL^–1^ and no change in MBC was observed in the case of E. coli. On the other hand, GR-MOF-23 presented no bactericidal effect against both strains at all of the tested concentrations.

The MICs of other bisphosphonates in different bacterial strains for both Gram-positive and Gram-negative bacteria have been documented in the literature,^31^ being much lower than those reported in this study (*e*.*g*., against S. aureus: 500, >1.26, and >400 μg·mL^–1^ for ibandronate, pamidronate, and zoledronate; respectively vs 16,500 and 7000 μg·mL^–1^ for GR-MOF-23 and GR-MOF-24; respectively). These differences may be related to the fact that the BPs reported in this study are part of hybrid materials; meanwhile, the values reported in the literature belong to directly dissolved BPs different from MA. Also, Sato et al. already found that the inhibitory concentrations of bone resorption are much higher in the *in vivo* model compared to the *in vitro* model to achieve cellular apoptosis.^32^ On the other hand, the mechanism of antimicrobial action of BPs has been already discussed in the literature.^33,34^ For non-nitrogenous BPs, as the medronate ligand used on the prepared materials, it is described that the antibacterial effect is mainly due to the aminoacyl-tRNA synthetases as a consequence of their resemblance to inorganic pyrophosphate (PPi). These enzymes integrate the medronate into adenosine to create methylene-containing ATP analogues, as occurs in osteoclasts. Specifically, Barbosa et al. defined that the MA, once inside the bacterium metabolically, aggregates to an ATP analogue called adenosine 5′-*O*-(2,3-methylene triphosphate) (AppCH_2_p), which differs from β,γ-pyrophosphate (P–O–P) ATP only in the presence of the P–C–P moiety of medronate. These nonhydrolyzable bonds generate an accumulation of AppCH_2_p inside the cell, blocking the adenosine translocase enzyme and causing apoptosis.^33^ Finally, the antibacterial effect could be also associated with an interaction of BPs with the phospholipids of the bacterial membrane. Phosphonate groups have a high affinity for hydroxyl groups and metal ions that are present in the phospholipids of the bacterial membrane, allowing for the binding of the PBs to the phospholipids. This binding changes the composition and rearranges the lipidic bilayer, modifying their fluidity and stability and thus creating transition areas in the membrane that may be more susceptible to pore formation. Therefore, these data suggest that the value required to achieve an antibacterial effect may be similar to that for bacterial cell death.

In short, these compounds could have the potential to form cements or implant coatings aimed at fracture repair. Additionally, it has been described in the literature as an antibiofilm effect of the BPs, either alone or in combination with other compounds.^35,36^ Hiltunen et al. found that the presence of BPs combined with bioactive glass or alone prevented the creation of a biofilm of two *Staphylococcus* strains.^35^ Although those compounds have great potential against bacteria, it must be taken into account that there are other data indicating an increase in bacterial adhesion to surfaces with PBs.^37,38^ Ganguli et al. studied bacterial adhesion on apatite coated with BPs, and they found that it was enhanced or did not depend on the compound used. In their work, they pointed out the importance of the electrostatic interaction between bacteria and the BP: the increased bacterial adhesion reported on apatite with pamidronate was due to the presence of amino groups in the compound, which would attract bacteria through direct interaction with the surface proteins. Instead, when the coating was clodronate, adhesion was slightly reduced.^37^ It is also interesting to consider the relatively low release of calcium estimated in our study, since the presence of calcium is related to the promotion of biofilm formation, increasing its stability.^39^ This fact could explain why the GR-MOF-23 compound shows less activity than GR-MOF-24. The MIC of GR-MOF-24 is lower than that of GR-MOF-23, and GR-MOF-23 does not show any MBC.

The antibacterial activity displayed by these novel compounds adds value to their potential pharmacological activity in the field of osteoporosis. Especially a direct application can be found in bone fillings or coatings where the presence of a high concentration of the compound would guarantee effective protection against infections.

## 4 Conclusions

Two novel bioactive MOF materials (denoted as GR-MOF-23 and GR-MOF-24) based on Ca^2+^ and medronic acid have been successfully synthesized, and their structures are fully characterized. The 2D crystal structure of GR-MOF-23 comprises two calcium cations with markedly different coordination environments linked by dianionic medronates. Further, when GR-MOF-23 is dried, a novel 3D crystal structure (GR-MOF-23-dried) is formed. The GR-MOF-24 crystal structure comprises Ca­(II) cations with the CaO_7_ coordination sphere linked to two monodentated phosphonate groups, two asymmetric chelating phosphonate groups, and methanol.

Both materials can be considered calcium reservoirs, as they can release Ca^2+^ in PBS, achieving 38.0 ± 2.8 and 35.8 ± 3.9% release of calcium after 1 week. Further, the solid residue was identified as apatite and calcium phosphate, which might facilitate the formation of bone apatite and collagen. Remarkably, both materials inhibit the growth of E. coli and S. aureus, while only GR-MOF-24 presented a bactericidal effect against S. aureus. These novel materials offer interesting possibilities in antibacterial surface development in prostheses and in the treatment of bone fractures.

## Supplementary Material



## Notes

Further materials characterization: FTIR spectra, TGA, and single-crystal X-ray diffraction refinement and crystallographic data (PDF)
