# Supplementary material for: Calcium Medronate-Based Metal–Organic Frameworks as Multifunctional Biomaterials
Source: Cryst Growth Des. 2025 Feb 20;25(5):1415–22. doi: 10.1021/acs.cgd.4c01478 (PMC12150673; doi:10.1021/acs.cgd.4c01478)
Supplement: Supplementary file 1 [file cg4c01478_si_001.pdf]

Calcium Medronate-based Metal-Organic Frameworks as multifunctional biomaterials

Pablo Salcedo-Abraira,[a] María Fernández-Grajera,[b] Francisco A. Guerrero-Román,[a] Antonio Rodríguez-Diéguez,[a] Veronica Luque-Agudo,[c] María Luisa González-Martín,[d] Amparo M. Gallardo-Moreno,[d,*] Sara Rojas [a,*]

[a] Department of Inorganic Chemistry, University of Granada, Avda. Fuente nueva s/n, 18071 Granada, Spain. E-mail:

[b] Center for Networked Biomedical Research on Bioengineering, Biomaterials and Nanomedicine (CIBER-BBN), Badajoz 06006, Spain

[c] Department of Natural Systems and Resources, School of Forest Engineering and Natural Resources, Polytechnic University of Madrid, C/ José Antonio Novais, 10, 28040 Madrid, Spain.

[d] Department of Applied Physics, Faculty of Sciences, University of Extremadura, Badajoz 06006, Spain

Supporting information

Table of contents

S1. Materials characterization……………………………….S2

S2. Stability studies…………………………………………….S6

S3. Antibacterial test…………………………………………..S7

S4. References…………………………………………………..S8

S1. Materials characterization.

Single-crystal X-ray diffraction refinement and crystallographic data.

Data collection: Single crystal X-ray diffraction data were collected at 150 K and room temperature on a Bruker D8 Venture Mo Kα (λ = 0.71073 Å) equipped with a PHONTON 3 detector. Data were collected and processed using APEX IV software. Adsorption correction was applied using SADABS software by empirical methods measuring symmetry equivalent reflections at different azimuthal angles. The structure was solved using the SHELXT program and refined using least squares refinement methods on all F^2 values as implemented within SHELXL.[1,2] Both SHELXT and SHELXL were operated through the Olex2 (v1.5) interface.[3] All non-hydrogen framework atoms were refined with anisotropic displacement parameters and hydrogens were placed in the geometrical theoretical positions and given fixed isotropic displacement parameters.

Crystallographic data for GR-MOF-23, GR-MOF-23_dry and GR-MOF-24 have been deposited at the in the CCDC with 2383637-2383639 numbers and details of the data collections and crystal structure refinement are given in the crystallographic section of the supplementary information for this paper. These data can be obtained free of charge via www.ccdc.cam.ac.uk/data_request/cif, or by emailing data_request@ccdc.cam.ac.uk, or by contacting The Cambridge Crystallographic Data Centre, 12 Union Road, Cambridge, CB2 1EZ, UK; fax: +44 1223 336033. e-mail: deposit@ccdc.cam.ac.uk or http://www.ccdc.cam.ac.uk).

Table S1. Crystallographic data parameters

Identification code	GR-MOF-23	GR-MOF-23_dry	GR-MOF-24
CCDC number	2383637	2383638	2383639
Empirical formula	$C_2H_{12}Ca_2O_{14}P_4$	$CH_4CaO_6P_2$	$C_2H_8CaO_7P_2$
Formula weight	464.16	214.06	246.10
Temperature/K	296.15	296.15	100.00
Crystal system	triclinic	monoclinic	monoclinic
Space group	*P*-1	*C*2/*c*	$P2_1/n$
a/Å	7.1189(6)	7.8014(4)	6.9615(4)
b/Å	10.3920(11)	8.0466(4)	6.7215(4)
c/Å	11.0416(10)	9.6305(4)	16.6674(11)
α/°	65.229(4)	90	90
β/°	89.556(3)	102.560(2)	99.833(2)
γ/°	83.459(3)	90	90
Volume/Å^3	736.14(12)	590.08(5)	768.44(8)
Z	2	4	4
ρ_{calc}g/cm^3	2.094	2.410	2.127
μ/mm^{-1}	1.278	1.572	1.231
F(000)	472.0	432.0	504.0
Crystal size/mm^3	0.5 × 0.01 × 0.01	0.12 × 0.1 × 0.08	0.18 × 0.15 × 0.11
Radiation	MoKα (λ = 0.71073)	MoKα (λ = 0.71073)	MoKα (λ = 0.71073)
2Θ range for data collection/°	4.066 to 55.1	7.368 to 54.99	4.96 to 57.404

Index ranges	-9 ≤ h ≤ 8, -13 ≤ k ≤ 13, -14 ≤ l ≤ 14	-10 ≤ h ≤ 10, -10 ≤ k ≤ 9, -12 ≤ l ≤ 12	-8 ≤ h ≤ 9, -7 ≤ k ≤ 9, -22 ≤ l ≤ 22
Reflections collected	17208	4288	12025
Independent reflections	3394 [R_{int} = 0.1075, R_{sigma} = 0.0781]	681 [R_{int} = 0.0575, R_{sigma} = 0.0320]	1982 [R_{int} = 0.0767, R_{sigma} = 0.0491]
Data/restraints/parameters	3394/0/205	681/0/49	1982/4/115
Goodness-of-fit on F^2	1.019	1.096	1.158
Final R indexes [I>=2σ (I)]	R_1 = 0.0437, wR_2 = 0.0967	R_1 = 0.0232, wR_2 = 0.0607	R_1 = 0.0521, wR_2 = 0.1162
Final R indexes [all data]	R_1 = 0.0728, wR_2 = 0.1114	R_1 = 0.0269, wR_2 = 0.0620	R_1 = 0.0863, wR_2 = 0.1461
Largest diff. peak/hole / e $Å^{-3}$	0.54/-0.50	0.30/-0.34	0.92/-0.81

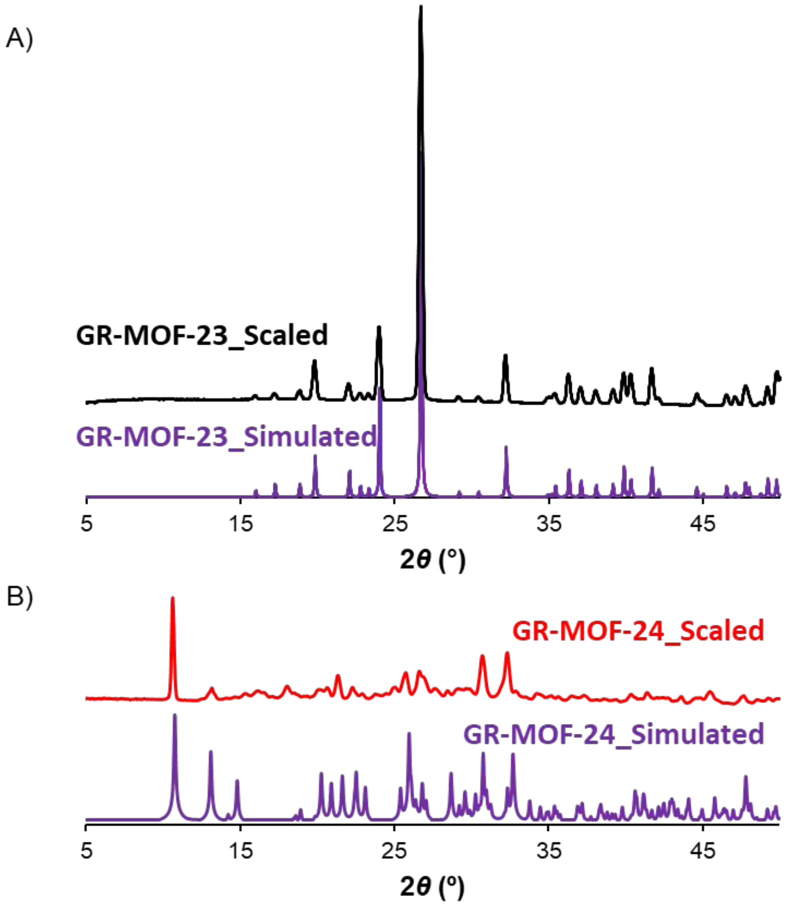

Figure S1. Experimental XRPD patters of GR-MOF-23 (A) and GR-MOF-24 (B) compared with the simulated ones form SCXRD.

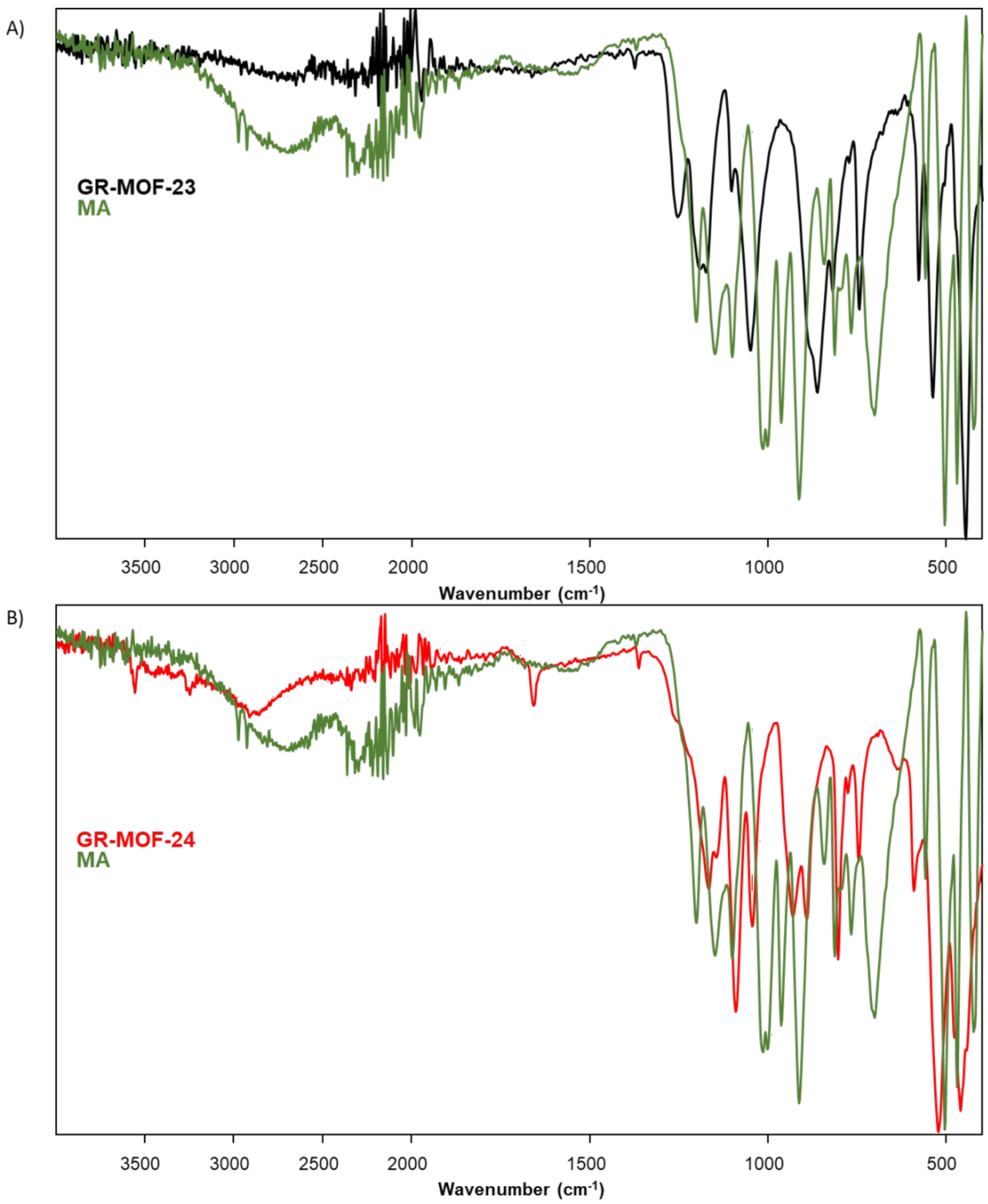

Figure S2. FTIR spectra of (A) GR-MOF-23 and (B) GR-MOF-24 compared with the free linker.

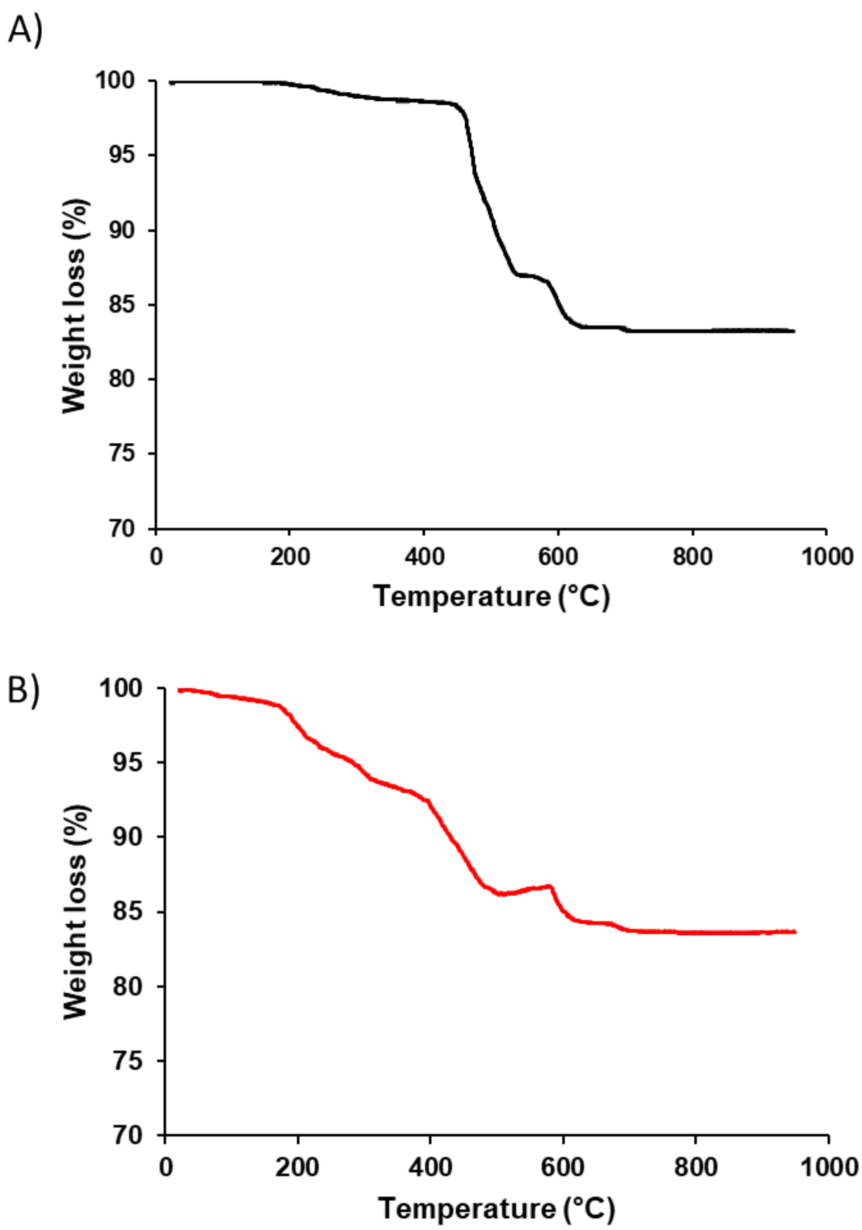

Figure S3. Thermogravimetric analysis of (A) GR-MOF-23 and (B) GR-MOF-24.

S2. Stability studies.

The chemical and structural stability of GR-MOF-23 and GR-MOF-24 was determined in phosphate buffer saline (PBS) at 37 °C by measuring the release of Ca by ICP-OES. 20 mg of each compound were suspended in 20 mL of PBS under bidimensional stirring for 7 days. At different incubation times (0, 0.25, 0.5, 1, 2, 4, 8, 24, 48, 120 and 168 h) an aliquot of 10 mL was extracted and the same volume of PBS at 37 °C was added to the suspension in a way to keep sink conditions. All kinetic studies were carried out in triplicate (*n* =3). At the end of the experiment, the remaining solids were analyzed by XRPD in order to check the crystallinity of the structure.

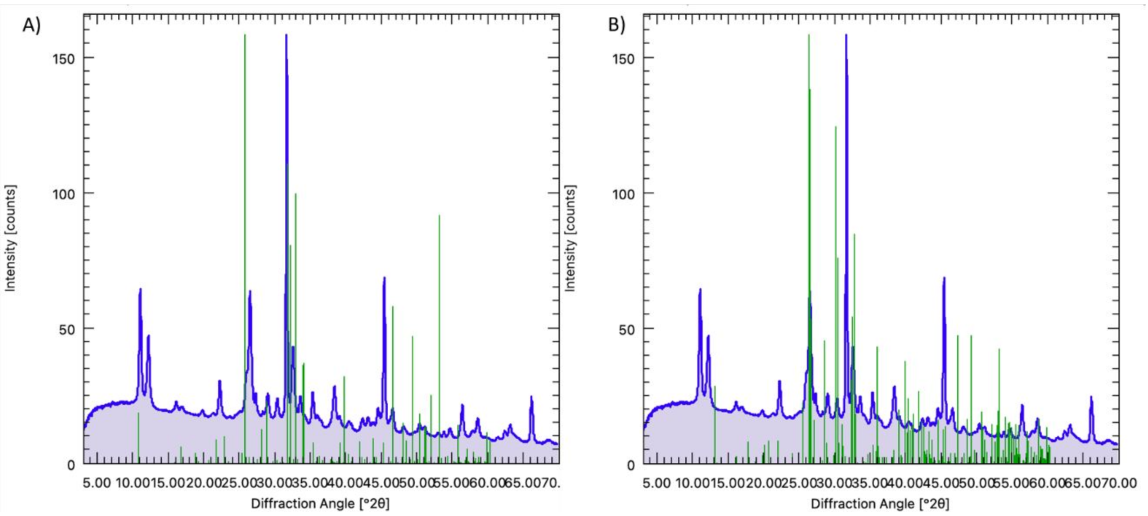

Figure S4. Powder X-ray diffraction (XRD) patterns of GR-MOF-24 after been suspended in a phosphate buffer solution (PBS) at 37 °C for 1 week, and its potential identified residual mixture of (A) apatite and (B) calcium phosphate.

S3. Antibacterial test

Table S2. Main results of the antibacterial activity test of GR-MOF-23 and GR-MOF-24.

	**GR-MOF-23**		**GR-MOF-24**	
	MIC ($mg{\cdot}mL^{-1}$)	MBC ($mg{\cdot}mL^{-1}$)	MIC ($mg{\cdot}mL^{-1}$)	MBC ($mg{\cdot}mL^{-1}$)
S. aureus	16.5	-	7	16
E. coli	16.5	-	7.5	-

S4. References.

(1) Sheldrick, G. M. Crystal Structure Refinement with SHELXL. *Acta Crystallogr C Struct Chem* **2015**, *71* (1), 3–8. https://doi.org/10.1107/S2053229614024218.

(2) Sheldrick, G. M. SHELXT – Integrated Space-Group and Crystal-Structure Determination. *Acta Crystallogr A Found Adv* **2015**, *71* (1), 3–8. https://doi.org/10.1107/S2053273314026370.

(3) Dolomanov, O. V.; Bourhis, L. J.; Gildea, R. J.; Howard, J. A. K.; Puschmann, H. OLEX2 : A Complete Structure Solution, Refinement and Analysis Program. *J Appl Crystallogr* **2009**, *42* (2), 339–341. https://doi.org/10.1107/S0021889808042726.
